# Interim analysis for post-marketing surveillance of dabrafenib and trametinib combination therapy in Japanese patients with unresectable and metastatic melanoma with *BRAF* V600 mutation

**DOI:** 10.1007/s10147-020-01737-3

**Published:** 2020-07-22

**Authors:** Yasutomo Teshima, Minako Kizaki, Ryohei Kurihara, Ryosuke Kano, Miki Harumiya

**Affiliations:** 1 Re-Examination, Patient Safety Japan Re-Examination, Regulatory Office Japan, Novartis Pharma K.K., Toranomon Hills Mori Tower, 1-23-1, Toranomon, Minato-ku, Tokyo, 105-6333 Japan; 2 PVO Japan, Patient Safety Japan, Regulatory Office Japan, Novartis Pharma K.K., Toranomon Hills Mori Tower, 1-23-1, Toranomon, Minato-ku, Tokyo, 105-6333 Japan; 3 Biostatistics, Clinical Development, Novartis Pharma K.K., Toranomon Hills Mori Tower, 1-23-1, Toranomon, Minato-ku, Tokyo, 105-6333 Japan; 4 Solid Tumor Medical Franchise Department, Oncology Medical Affairs Department, Novartis Pharma K.K., Toranomon Hills Mori Tower, 1-23-1, Toranomon, Minato-ku, Tokyo, 105-6333 Japan; 5 Clinical Development of Solid Tumor Oncology Group, Clinical Development Department, Novartis Pharma K.K., Toranomon Hills Mori Tower, 1-23-1, Toranomon, Minato-ku, Tokyo, 105-6333 Japan

**Keywords:** Dabrafenib, Trametinib, Real-world, Melanoma, *BRAF* mutation, Japanese

## Abstract

**Purpose:**

To investigate the safety and efficacy of dabrafenib and trametinib combination therapy for *BRAF* V600 mutation-positive unresectable and metastatic melanoma in over 100 Japanese patients of a real-world clinical setting.

**Patients:**

The surveillance period of interim post-marketing surveillance (PMS) analysis was from June 2016 to November 2018, and 112 patients with unresectable and metastatic *BRAF* V600 melanoma who received dabrafenib and trametinib were enrolled.

**Results:**

The safety analysis set included 112 patients whom almost all patients had stage IV disease (*n* = 97, 86.61%) with an Eastern Cooperative Oncology Group performance status 0 or 1 (*n* = 102, 91.07%), and mean (standard deviation) lactate dehydrogenase level was 354.3 (456.4) U/L (*n* = 105) at baseline. Median daily dose of dabrafenib was 300.0 mg/day (118–300), and median daily dose of trametinib was 2.00 mg/day (1.0–4.0). Adverse drug reactions (ADRs) were reported in 84 patients (75%), and common ADRs (incidence ≥ 5%) were pyrexia (*n* = 49, 43.75%), hepatic function abnormal (*n* = 11, 9.82%), rash and blood creatine phosphokinase increased (*n* = 9 each, 8.04%), and erythema nodosum (*n* = 6, 5.36%). Majority of ADRs reported in this study were consistent with that reported in previous trials. In the efficacy analysis set of 110 patients, the objective response rate was 55.45% (95% confidence interval 45.67–64.93%), and median progression-free survival was 384.0 days (251.0 days-not reached).

**Conclusions:**

No new safety or efficacy concerns were observed in this interim PMS analysis in Japanese patients with unresectable and metastatic melanoma with *BRAF* gene mutation who received dabrafenib and trametinib combination therapy.

**Electronic supplementary material:**

The online version of this article (10.1007/s10147-020-01737-3) contains supplementary material, which is available to authorized users.

## Introduction

Melanomas are malignant tumors arising from pigment cells/melanocytes [1]. Although melanomas are mostly of cutaneous origin, they can also occur at a low frequency in various extra-cutaneous sites, where non-cutaneous melanocytes are present, including the respiratory, gastrointestinal, and genitourinary sites [1]. Studies have reported ethnic differences in the incidence of melanoma. The disease is common in Caucasians, with more than 76,000 new cases and 9000 deaths reported in the US in 2012 [2], whereas the disease is less common in Asians, including the Japanese, with approximately 1–2 new cases reported per 100,000 people per year [3] and about 4000 patients reported with melanoma in 2016 in Japan [4].

Historically, metastatic melanoma has been considered as one of the most therapeutically challenging malignancies [5, 6]. However, since the discovery of *BRAF* V600 somatic missense mutations in approximately 50% of malignant melanomas and at a lower frequency in a wide range of human cancers [7], there has been remarkable progress in the development of targeted therapies for unresectable and metastatic melanoma, with high response rates [5, 6]. *BRAF* mutations result in constitutive activation of the BRAF kinase protein, thereby promoting hyperactivation of the mitogen-activated protein kinase (MAPK) signaling pathway, which includes mitogen-activated protein kinase kinase (MEK), an essential regulator of cell proliferation and survival [8].

Dabrafenib is a potent and selective inhibitor of BRAF kinase activity [9], and trametinib is a reversible, highly selective allosteric inhibitor of MEK1/MEK2 activation [10, 11]. In pivotal, global phase 3 studies, the combination of dabrafenib and trametinib demonstrated statistically significant and clinically relevant improvements in progression-free survival (PFS) and overall survival in patients with *BRAF* V600 mutation–positive melanoma, which established this combination therapy as a standard treatment option for *BRAF* V600 mutation–positive melanoma [12]. Dabrafenib and trametinib as monotherapy or as combination therapy have been approved in the US and Europe for the indication of unresectable or metastatic melanoma with *BRAF* V600 mutations. The frequency of *BRAF* V600 mutations was reported to be lower in the Japanese than in Caucasians [13]. A phase 1/2 study in Japanese patients with *BRAF* V600 mutation–positive advanced cutaneous melanoma demonstrated that the efficacy and pharmacokinetic properties of the dabrafenib and trametinib combination in Japanese patients were comparable with those observed in global studies [14]. In Japan, dabrafenib monotherapy and combination therapy of dabrafenib and trametinib were approved for use in the treatment of patients with *BRAF* mutation–positive unresectable malignant melanoma in March 2016.

However, the number of Japanese patients in the clinical study for the new drug application was small. As a result, a post-marketing surveillance (PMS) of dabrafenib and trametinib in Japanese patients with unresectable melanoma with *BRAF* mutation was initiated in June 2016 to determine the safety and efficacy of dabrafenib and trametinib in a Japanese clinical setting. The PMS is being conducted on the condition that all Japanese melanoma patients treated with dabrafenib- and/or in clinical practice may be enrolled. Here, we report the interim results of this ongoing PMS based on the analysis of data collected until November 2018 (data cutoff).

## Materials and methods

### Study design and patients

This was an interim analysis of a PMS being conducted in Japan to collect information on the safety and efficacy of dabrafenib and trametinib in clinical practice. Target patients for this investigation were all patients with *BRAF* V600 mutation-positive melanoma who were administered dabrafenib and/or trametinib in Japan since June 2016 to September 2017 (all-case investigation). Patients with melanoma who were administered dabrafenib and trametinib as an adjuvant therapy after surgery were excluded from the PMS. Patients treated with dabrafenib or trametinib monotherapy were enrolled but excluded from the safety and efficacy analysis sets in the PMS. The observation period was 1 year.

The PMS is an observational survey being conducted by Novartis Pharma K.K. in accordance with the ministerial ordinance on Good Post-marketing Study Practice in Japan, which determines that informed consent is unnecessarily required for conducting the PMS in Japan.

### Study assessments

Demographic and baseline data were collected for each patient at enrollment or at first data collection in the PMS, which included information on the sex, date of birth (or age), patient identification number, reason for prescription of dabrafenib and/or trametinib, start date of treatment, visit category, date of diagnosis of primary disease, history of adverse drug reactions (ADRs), history of allergy, cancer stage, Eastern Cooperative Oncology Group performance status (ECOG PS) at the start of surveillance, past and current medical conditions at the start of treatment, prior therapy for primary disease, and pregnancy status (women only).

The status of dabrafenib and/or trametinib administration was monitored by recording the start date of treatment, daily dose, treatment duration, changes to dosing regimen (date of change and dose) and its reason, and date of dose discontinuation or interruption and its reason.

The safety endpoint was the incidence of adverse events (AEs) and ADRs, defined as AEs in which a causal relationship to dabrafenib and/or trametinib was not denied by the investigator. The seriousness of observed AEs and ADRs was also assessed by the physicians according to the International Conference on Harmonisation standards. Observed AEs and ADRs were tabulated by system organ class (SOC) and preferred term (PT) using the Medical Dictionary for Regulatory Activities/Japanese (MedDRA/J) version 21.1. Each SOC was tabulated as the number of patients who experienced events, and each PT was tabulated as the number of events. If events of the same PT occurred more than once in the same patient, they were counted as 1 event for tabulation purposes. Observed AEs and ADRs were graded using the National Cancer Institute Common Terminology Criteria for Adverse Events version 4.0. Safety specifications in this PMS included the incidences of the following events: cardiac disorders, hepatic impairment, pyrexia, eye disorders, cutaneous squamous cell carcinoma, secondary malignancies other than cutaneous squamous cell carcinoma, and rhabdomyolysis.

The efficacy endpoints were anti-tumor effect based on the physician’s overall assessment and PFS. For the assessment of anti-tumor effect, the physicians evaluated the best response, including complete response (CR) or partial response (PR), during the observation period in accordance with the Response Evaluation Criteria in Solid Tumors (RECIST) version 1.1 [15]. The date of evaluation was recorded, and when disease progression or death was confirmed, that date was also recorded in the survey sheet.

### Statistical analysis

All statistical analyses were performed using descriptive statistics. The mean [standard deviation (SD)] and median [minimum (min) and maximum (max)] were calculated for continuous variables, and the frequency and proportion were calculated for categorical variables. The objective response rate (ORR) based on best response during the observation period was defined as the percentage (%) of patients with CR or PR in accordance with RECIST version 1.1 [15]. The numbers and percentages of patients were also tabulated by adjudicated tumor response. Patients assessed as not evaluable (NE) were considered as non-responders and included in the denominator to calculate response rates. For response rates, the 95% confidence interval (CI) was estimated by the Clopper–Pearson method. PFS in patients treated with dabrafenib and trametinib was descriptively summarized using the Kaplan–Meier method. All statistical analyses were performed using SAS version 9.1.3 and 9.3 (SAS Institute Japan Ltd., Tokyo, Japan).

## Results

### Analysis data set

In the PMS of dabrafenib and trametinib, 372 patients were registered at 118 medical sites across Japan since the start of the survey (June 2016), and data of 118 patients were locked until November 2018 (data cutoff). The safety analysis set included 112 patients, after excluding 6 patients who were administered dabrafenib and trametinib combination therapy within 2 week duration following the exclusion criteria in the protocol. The efficacy analysis set included 110 patients from the safety analysis set, after excluding 2 patients for whom efficacy results were missing.

### Baseline clinical characteristics of patients

Of the 112 patients in the safety analysis set, 52.68% were male. The mean (SD) age was 59.10 (15.02) years, and most patients were less than 75 years of age (95 patients, 84.82%). The main baseline clinical characteristics were stage IV disease (97 patients, 86.61%) and ECOG PS 0 or 1 (102 patients, 91.07%). Immune checkpoint inhibitor therapies were received by 45 patients (40.18%) before treatment initiation of dabrafenib and trametinib (Table 1). Mean (SD) lactate dehydrogenase [LDH] level was 354.3 (456.4) U/L (*n* = 105).

**Table 1:** Baseline clinical characteristics of the safety analysis set (112 patients)

Characteristics	Category, summary statistics	*n* (%)
Sex	Male	59 (52.68)
	Female	53 (47.32)
Pregnancy (women only)	No	52 (98.11)
	Yes	0 (–)
	Unknown	1 (1.89)
Age (year)	*n*	112
	Mean (SD)	59.1 (15.02)
	Median (min–max)	60.5 (19–86)
Duration of melanoma (months)	*n*	101
	Mean (SD)	24.7 (24.43)
	Median (min–max)	18.0 (0–123)
Cancer stage at baseline	0	0 (–)
	IA	0 (–)
	IB	0 (–)
	IIA	0 (–)
	IIB	1 (0.89)
	IIC	0 (–)
	IIIA	1 (0.89)
	IIIB	2 (1.79)
	IIIC	10 (8.93)
	IV	97 (86.61)
	Unknown	1 (0.89)
ECOG PS at baseline	0	72 (64.29)
	1	30 (26.79)
	2	6 (5.36)
	3	4 (3.57)
	4	0 (–)
Prior therapy for primary disease	No	42 (37.50)
	Yes	70 (62.50)
Dacarbazine	No	84 (75.00)
	Yes	28 (25.00)
Nimustine	No	94 (83.93)
	Yes	18 (16.07)
Vincristine	No	97 (86.61)
	Yes	15 (13.39)
Interferon	No	70 (62.50)
	Yes	42 (37.50)
Interleukin-2	No	112 (100.00)
	Yes	0 (–)
Nivolumab	No	71 (63.39)
	Yes	41 (36.61)
Vemurafenib	No	73 (65.18)
	Yes	39 (34.82)
Ipilimumab	No	96 (85.71)
	Yes	16 (14.29)
Pembrolizumab	No	112 (100.00)
	Yes	0 (–)
Others	No	107 (95.54)
	Yes	5 (4.46)
Immuno-oncology therapy^a^	No	67 (59.82)
	Yes	45 (40.18)

*ECOG PS* Eastern Cooperative Oncology Group performance status; *SD* standard deviation

^a^Immune-Oncology therapy defined as treatment for nivolumab, pembrolizumab, and ipilimumab in this study

### Status of dabrafenib and trametinib therapy

Of 112 patients in the safety analysis set, 69 patients (61.61%) discontinued dabrafenib and trametinib therapy, and the most frequent reasons for discontinuation were AEs in 41 patients (59.42%) and an inadequate clinical response in 32 patients (46.38%) (which included some overlap).

All patients were treated with the approved dose (300 mg/day or less) of dabrafenib, and the mean (SD) dose was 267.80 (51.72) mg/day. Most patients (97.32%) were treated with the approved dose (2 mg/day or less) of trametinib, and the mean (SD) dose was 1.93 (0.387) mg/day. Mean (SD) duration of the combination therapy was 198.6 (128.11) days (Table 2).

**Table 2:** Status of dabrafenib and trametinib therapy

Factor	Summary statistics	Safety analysis set
Overall	*n*	112
Dabrafenib administration status		
Daily dose (mg/day)	*n*	112
	Mean (SD)	267.8 (51.72)
	Median (min–max)	300.0 (118–300)
Trametinib administration status		
Daily dose (mg/day)	*n*	112
	Mean (SD)	1.93 (0.387)
	Median (min–max)	2.00 (1.0–4.0)
Duration of the combination therapy of dabrafenib and trametinib (day)	*n*	112
	Mean (SD)	198.6 (128.11)
	Median (min–max)	156.0 (15–364)
	Total duration (person-year)	61.1

*SD* standard deviation

### Incidence of AEs and ADRs

Of the 112 patients in the safety analysis set, 97 patients (86.61%) experienced AEs, and the most common AEs (incidence ≥ 10%) were pyrexia in 50 patients (44.64%), disease progression in 37 patients (33.04%), and hepatic function abnormal in 12 patients (10.71%). Serious AEs occurred in 51 patients (45.54%), and the most common serious AEs (incidence ≥ 5%) were disease progression in 28 patients (25.00%), pyrexia in 11 patients (9.82%), and metastases to central nervous system in 6 patients (5.36%) (Table 3).

**Table 3:** Incidence of AEs and ADRs (reported in ≥ 5 patients as AEs)

PT	Safety analysis set (*n* = 112)					
	AEs of any grade	AEs of grade ≥ 3	Serious AEs^a)^	ADRs	ADRs of grade ≥ 3	Serious ADRs
	*n* (%)	*n* (%)	*n* (%)	*n* (%)	*n* (%)	*n* (%)
Total	97 (86.61)	44 (39.29)	51 (45.54)	84 (75.00)	27 (24.11)	25 (22.32)
Pyrexia	50 (44.64)	8 (7.14)	11 (9.82)	49 (43.75)	8 (7.14)	10 (8.93)
Disease progression	37 (33.04)	13 (11.61)	28 (25.00)	2 (1.79)	1 (0.89)	1 (0.89)
Hepatic function abnormal	12 (10.71)	1 (0.89)	2 (1.79)	11 (9.82)	1 (0.89)	1 (0.89)
Rash	9 (8.04)	0 (–)	0 (–)	9 (8.04)	0 (–)	0 (–)
Blood creatine phosphokinase increased	9 (8.04)	1 (0.89)	1 (0.89)	9 (8.04)	1 (0.89)	1 (0.89)
Malaise	7 (6.25)	1 (0.89)	1 (0.89)	5 (4.46)	1 (0.89)	1 (0.89)
Metastases to central nervous system	6 (5.36)	2 (1.79)	6 (5.36)	0 (–)	0 (–)	0 (–)
Diarrhoea	6 (5.36)	2 (1.79)	2 (1.79)	4 (3.57)	1 (0.89)	1 (0.89)
Erythema nodosum	6 (5.36)	0 (–)	0 (–)	6 (5.36)	0 (–)	0 (–)
Rhabdomyolysis	5 (4.46)	3 (2.68)	3 (2.68)	5 (4.46)	3 (2.68)	3 (2.68)

MedDRA/J version 21.1

^a)^ Two events were excluded from the analysis, since their seriousness were unknown

*ADR* adverse drug reaction; *AE* adverse event; *MedDRA/J* Medical Dictionary for Regulatory Activities/Japanese; *PT* preferred term

ADRs were reported in 84 patients (75.00%), and common ADRs (incidence ≥ 5%) were pyrexia in 49 patients (43.75%), hepatic function abnormal in 11 patients (9.82%), rash and blood creatine phosphokinase increased in 9 patients each (8.04%), and erythema nodosum in 6 patients (5.36%). ADRs of grade ≥ 3 were reported in 27 patients (24.11%), and the most common ADR of grade ≥ 3 (incidence ≥ 5%) was pyrexia in 8 patients (7.14%) (Table 3). The outcomes of these ADRs were as follows: death (*n* = 1 patient; due to disease progression); not recovered (*n = *4 patients with pyrexia and *n* = 1 patient each with iron deficiency anemia, uveitis, dermatitis acneiform, blood lactate dehydrogenase increased, and putamen hemorrhage); unknown status (*n* = 1 patient each with uveitis, disease progression, metastases to skin, and alopecia); and recovered or recovering (in all remaining patients with ADRs excluding the above events). Serious ADRs were reported in 25 patients (22.32%), and the most common serious ADR (incidence ≥ 5%) was pyrexia in 10 patients (8.93%). The outcomes of the serious ADRs were as follows: 1 patient died of disease progression; 2 patients did not recover from serious ADRs, 1 patient with blood LDH increased, and the other with putamen hemorrhage. Status of serious ADR outcome was unknown in 1 patient with uveitis. All other patients recovered or are recovering from all ADRs, excluding the above events. The incidence of ADRs between the 2 groups of patients with and without previous IO therapy were similar [32 patients (71.11%) and 52 patients (77.61%), respectively].

Overall, 25 deaths were reported, including 1 death for which a causal relationship with dabrafenib and trametinib could not be ruled out in this PMS. The reason for death was disease progression.

Safety specifications were reported in 63 patients (56.25%), and the most commonly reported ADRs of safety specifications (incidence ≥ 3%) were pyrexia in 49 patients (43.75%), hepatic impairment in 16 patients (14.29%), and eye disorders in 6 patients (5.36%) (Table 4). The time to onset and time to outcome evaluation for ADRs of safety specifications are shown in Supplementary Table 1. The median (min–max) time to onset for the most commonly observed event, pyrexia, was 11.0 days (1–125 days) and time to outcome for pyrexia was 17.0 days (2–649 days) (Supplementary Table 1); pyrexia occurred median 1.0 time/patient (1–3 times/patient) among 49 patients who experienced this ADR. Treatment taken after the onset of ADRs of safety specifications (incidence ≥ 5%) and the outcomes are shown in Supplementary Table 2.

**Table 4:** Incidence of AEs and ADRs of safety specifications

Events of safety specifications	Safety analysis set (*N* = 112)		Outcomes of ADRs					
	AEs	ADRs	Recovered	Recovering	Not recovered	Recovered with sequelae	Death	Unknown
	*n* (%)	*n* (%)	*n* (%)	*n* (%)	*n* (%)	*n* (%)	*n* (%)	*n* (%)
Total	82 (73.21)	63 (56.25)	31 (49.21)	24 (38.10)	5 (7.94)	0 (–)	1 (1.59)	2 (3.17)
Pyrexia	50 (44.64)	49 (43.75)	29 (59.18)	16 (32.65)	4 (8.16)	0 (–)	0 (–)	0
Secondary malignancies other than cutaneous squamous cell carcinoma	40 (35.71)	2 (1.79)	0 (–)	0 (–)	0 (–)	0 (–)	1 (50.00)	1 (50.00)
Disease progression	37 (33.04)	2 (1.79)	0 (–)	0 (–)	0 (–)	0 (–)	1 (50.00)	1 (50.00)
Hepatic impairment	17 (15.18)	16 (14.29)	11 (68.75)	5 (31.25)	0 (–)	0 (–)	0 (–)	0 (–)
Hepatic function abnormal	12 (10.71)	11 (9.82)	7 (63.64)	4 (36.36)	0 (–)	0 (–)	0 (–)	0 (–)
Aspartate aminotransferase increased	2 (1.79)	2 (1.79)	1 (50.00)	1 (50.00)	0 (–)	0 (–)	0 (–)	0 (–)
Alanine aminotransferase increased	2 (1.79)	1 (0.89)	0 (–)	1 (100.00)	0 (–)	0 (–)	0 (–)	0 (–)
Liver disorder	1 (0.89)	1 (0.89)	1 (100.00)	0 (–)	0 (–)	0 (–)	0 (–)	0 (–)
Hepatic enzyme increased	1 (0.89)	1 (0.89)	1 (100.00)	0 (–)	0 (–)	0 (–)	0 (–)	0 (–)
Hepatic enzyme abnormal	1 (0.89)	1 (0.89)	1 (100.00)	0 (–)	0 (–)	0 (–)	0 (–)	0 (–)
Eye disorders	7 (6.25)	6 (5.36)	1 (16.67)	3 (50.00)	1 (16.67)	0 (–)	0 (–)	1 (16.67)
Uveitis	4 (3.57)	4 (3.57)	0 (–)	2 (50.00)	1 (25.00)	0 (–)	0 (–)	1 (25.00)
Visual acuity reduced	1 (0.89)	1 (0.89)	1 (100.00)	0 (–)	0 (–)	0 (–)	0 (–)	0 (–)
Chorioretinopathy	1 (0.89)	1 (0.89)	0 (–)	1 (100.00)	0 (–)	0 (–)	0 (–)	0 (–)
Rhabdomyolysis	5 (4.46)	5 (4.46)	2 (40.00)	3 (60.00)	0 (–)	0 (–)	0 (–)	0 (–)
Rhabdomyolysis	5 (4.46)	5 (4.46)	2 (40.00)	3 (60.00)	0 (–)	0 (–)	0 (–)	0 (–)
Myoglobin blood increased	1 (0.89)	1 (0.89)	0 (–)	1 (100.00)	0 (–)	0 (–)	0 (–)	0 (–)
Cardiac disorders	1 (0.89)	1 (0.89)	1 (100.00)	0 (–)	0 (–)	0 (–)	0 (–)	0 (–)
Cardiac failure	1 (0.89)	1 (0.89)	1 (100.00)	0 (–)	0 (–)	0 (–)	0 (–)	0 (–)
Cutaneous squamous cell carcinoma	0 (–)	0 (–)	0 (–)	0 (–)	0 (–)	0 (–)	0 (–)	0 (–)

MedDRA/J version 21.1

The percentage of patients for each outcome of ADR was calculated using total number of patients who experienced each ADR as denominator

*ADR* adverse drug reaction; *AE* adverse event; *MedDRA/J* Medical Dictionary for Regulatory Activities/Japanese

### Efficacy

In the efficacy analysis set of 110 patients, the response rate based on the physician’s overall assessment was evaluated in accordance with RECIST version 1.1. The ORR (CR + PR) evaluated as a best response during the observation period was 61/110 patients (55.45%, 95% CI 45.67–64.93%) (Table 5). The ORR was also calculated after dividing the patients into the following groups: patients with normal (≤ 222 U/L) or elevated (> 222 U/L) LDH levels and patients without or with prior IO therapy. The ORR per LDH was 27/44 patients (61.36%, 95% CI 45.50–75.64%) with normal LDH levels and 30/60 patients (50%, 95% CI 36.81–63.19%) with elevated LDH levels. In terms of prior IO therapy, the ORR was 35/66 patients (53.03%, 95% CI 40.34–65.44%) without prior IO therapy and 26/44 patients (59.09%, 95% CI 43.25–73.66%) with prior IO therapy (Table 5).

**Table 5:** Anti-tumor effect in the efficacy analysis set (110 patients)

Groups	CR	PR	Non-CR/Non-PD	SD	PD	NE	Objective response (CR + PR) *n* (%) [95% CI of objective response rate]
	*n* (%)	*n* (%)	*n* (%)	*n* (%)	*n* (%)	*n* (%)	
Total (*n* = 110)	8 (7.27)	53 (48.18)	1 (0.91)	18 (16.36)	22 (20.00)	8 (7.27)	61 (55.45) [45.67–64.93]
Normal LDH (*n* = 44)	6 (13.64)	21 (47.73)	1 (2.27)	7 (15.91)	7 (15.91)	2 (4.55)	27 (61.36) [45.50–75.64]
Elevated LDH (*n* = 60)	1 (1.67)	29 (48.33)	0	10 (16.67)	15 (25.00)	5 (8.33)	30 (50.00) [36.81–63.19]
Unknown (*n* = 6)	1 (16.67)	3 (50.00)	0	1 (16.67)	0	1 (16.67)	4 (66.67) [22.28–95.67]
Without prior IO therapy (*n* = 66)	5 (7.58)	30 (45.45)	1 (1.52)	11 (16.67)	13 (19.70)	6 (9.09)	35 (53.03) [40.34–65.44]
With prior IO therapy (*n* = 44)	3 (6.82)	23 (52.27)	0	7 (15.91)	9 (20.45)	2 (4.55)	26 (59.09) [43.25–73.66]

*CI* confidence interval, *CR* complete response, *IO* immuno-oncology, *LDH* lactate dehydrogenase, *NE* Not evaluable, *PD* progressive disease, *PR* partial response, *SD* stable disease

**Fig. 1:**
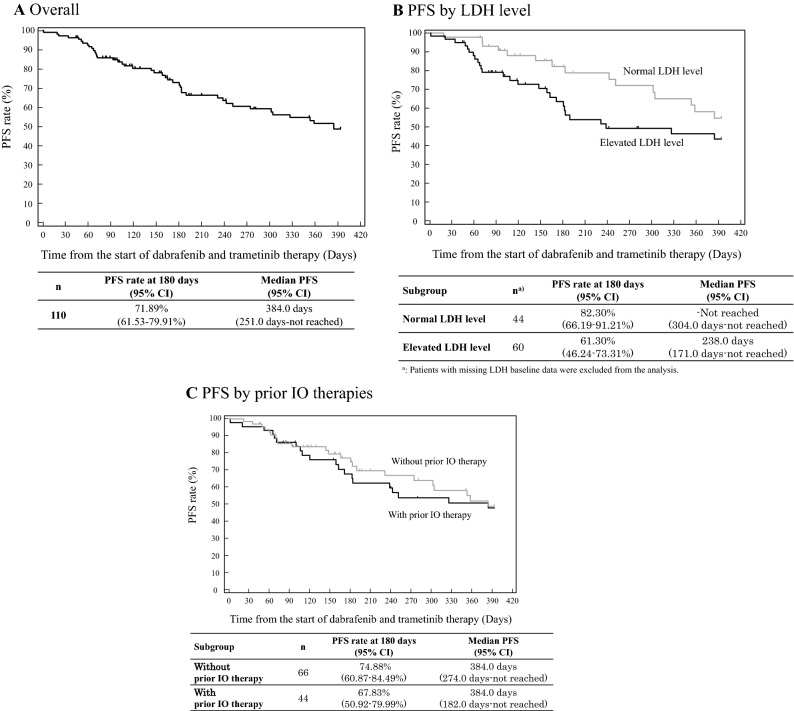
PFS estimated by Kaplan–Meier method. *CI* confidence interval, *IO* immuno-oncology, *LDH* lactate dehydrogenase, *PFS* progression-free survival

The PFS rate at 180 days was 71.89% (95% CI 61.53–79.91%) and median PFS was 384.0 days (251.00 days–not reached) (Fig. 1a). The PFS rates per LDH level were 82.30% (95% CI 66.19–91.21%) in the normal LDH level patient group and 61.30% (95% CI 46.24–73.31%) in the elevated LDH level patient group. The PFS rate in the patient groups without and with prior IO therapy was 74.88% (95% CI 60.87–84.49%) and 67.83% (95% CI 50.92–79.99%), respectively (Fig. 1b, c).

## Discussion

This is the first report describing prospectively analyzed, real-world data obtained from more than 100 Japanese patients with unresectable melanoma with *BRAF* mutation who received dabrafenib and trametinib. Due to ethnic differences in the incidences of melanoma, the disease is not common in the Japanese [3, 4] and Asians, unlike Caucasians and other people in western countries [2].

Of the 112 patients in the safety analysis set, 75.00% of patients reported ADRs, and the most common ADRs (incidence ≥ 5%) were pyrexia (43.75%), hepatic function abnormal (9.82%), rash and blood creatine phosphokinase increased (each in 8.04%), and erythema nodosum (5.36%). In the 5-year analysis of metastatic melanoma patients treated with dabrafenib plus trametinib in the COMBI-d and COMBI-v randomized trials, AEs, regardless of cause, occurred in 548 of 559 patients (98%); the most common AE was pyrexia (325 patients, 58%) [16]. Nearly all ADRs commonly observed in this investigation were previously reported events.

The incidence of ADRs of grade ≥ 3 was 24.11%, and the most common ADR of grade ≥ 3 (incidence ≥ 5%) was pyrexia (7.14%). Pyrexia is a well-documented potentially serious AE associated with dabrafenib and trametinib therapy. Clinicians should pay attention to this already-known risk of severe pyrexia when administering these drugs, and close monitoring is required.

In the PMS, safety specifications included the incidences of cardiac disorders, hepatic impairment, pyrexia, eye disorders, cutaneous squamous cell carcinoma, secondary malignancies other than cutaneous squamous cell carcinoma, and rhabdomyolysis, because these were clinically significant AEs observed in previous clinical trials of dabrafenib and trametinib. The incidence of safety specifications was 56.25%, and the commonly reported ADRs of safety specifications (incidence ≥ 5%) were pyrexia (43.75%), hepatic impairment (14.29%), and eye disorders (5.36%). The incidences, onset times, and outcomes of these ADRs varied with each item, as shown in Table 4 and Supplementary Tables 1 and 2. The occurrence of these ADRs should be continuously assessed in the clinical setting and relevant information should be provided to the physicians to establish more appropriate use of dabrafenib and trametinib.

The incidences of ADRs in patients with and without prior IO therapy were similar. Based on the present interim analysis of this PMS, no special measures are thought to be needed when dabrafenib and trametinib administration is considered for the patients with prior IO therapy.

In the efficacy analysis set of 110 patients, the ORR evaluated as a best response during the observation period was 55.45% (95% CI 45.67–64.93%) in the present investigation. In a global, phase 3, randomized, controlled trial, the overall response was reported in 144 patients among 211 patients treated with dabrafenib and trametinib (ORR 69%, 95% CI 62–75%) [12], which was similar to that of the present study.

LDH is an established prognostic biomarkers in advanced melanoma [17, 18]. In the present investigation, the ORR and PFS were analyzed after grouping the patients into 2 categories: patients with elevated LDH levels (> 222 U/L) and patients with normal LDH levels (≤ 222 U/L). The efficacy tended to be inferior in the patient group with high LDH levels versus patient group with normal LDH levels, which was consistent with previous reports [17, 18].

In the global, phase 3, randomized, controlled trial, the median PFS was reported to be 11.0 months (95% CI 8.0–13.9 months) in previously untreated patients [12]. Median PFS in the present investigation was 384.0 days (251.00 days–not reached), which was non-inferior to that of the global, phase 3 trial. We will continue to carefully evaluate the efficacy of dabrafenib and trametinib therapy in the PMS.

There were some limitations to this study. This was a single-arm surveillance study with no control group comprising patients not treated with dabrafenib and trametinib. The maximal observation period was 1 year. Additionally, the study design was an all-case investigation lacking patient selection criteria, which increased the representative patient population treated with dabrafenib and trametinib, reflective of the actual clinical setting. This study was conducted under Japanese-specific medical conditions including race, healthcare system, and drug utilization of anti-cancer drugs; therefore, the survey results may not be the same in other countries that have different medical conditions from Japan.

In conclusion, no new safety and efficacy concerns were observed in this interim analysis of PMS in Japanese patients with unresectable melanoma with *BRAF* mutation who received dabrafenib and trametinib in the real-world clinical setting. The study is ongoing to obtain more detailed information on the safety and efficacy of these drugs in clinical practice.

## Electronic supplementary material

Below is the link to the electronic supplementary material.

Supplementary material 1 (DOCX 67 kb)
