# Supplementary material for: Interim analysis for post-marketing surveillance of dabrafenib and trametinib combination therapy in Japanese patients with unresectable and metastatic melanoma with BRAF V600 mutation
Source: Int J Clin Oncol. 2020 Jul 22;25(10):1870–8. doi: 10.1007/s10147-020-01737-3 (PMC7498495; doi:10.1007/s10147-020-01737-3)
Supplement: Supplementary file 1 — Supplementary material 1 (DOCX 67 kb) [file 10147_2020_1737_MOESM1_ESM.docx]

**Supplementary Table 1. Time to onset and time to outcome for ADRs of safety specifications**

| **ADRs of safety specifications**  **Safety analysis set (N=112)** | **Incidences of ADRs** | | | **Onset time of ADRs (days)** | | | **Time to outcome for ADRs (days)** | | |
| --- | --- | --- | --- | --- | --- | --- | --- | --- | --- |
|  | **n** | **(%)** | | **n** | **Mean (SD)** | **Median (min-max)** | **n** | **Mean (SD)** | **Median (min-max)** |
| Pyrexia | 49 | | (43.75) | 49 | 19.8 (25.11) | 11.0 (1-125) | 49 | 76.0 (145.04) | 17.0 (2-649) |
| Hepatic impairment | 16 | | (14.29) | 16 | 53.4 (96.69) | 15.5 (9-392) | 16 | 34.7 (45.98) | 18.5 (5-190) |
| Eye disorders | 6 | | (5.36) | 6 | 85.7 (53.89) | 84.5 (5-162) | 6 | 100.7 (80.38) | 94.5 (22-237) |
| Rhabdomyolysis | 5 | | (4.46) | 5 | 76.4 (47.74) | 92.0 (15-125) | 5 | 36.6 (61.25) | 8.0 (7-146) |
| Secondary malignancies  other than cutaneous squamous cell carcinoma | 2 | | (1.79) | 2 | 227.0 (66.47) | 227.0 (180-274) | 2 | 70.5 (50.20) | 70.5 (35-106) |
| Cardiac disorders | 1 | | (0.89) | 1 | 41.0 | 41.0 (41-41) | 1 | 8.0 | 8.0 (8-8) |
| Cutaneous squamous cell carcinoma | 0 | | - | 0 | - | - | 0 | - | - |

*ADR* adverse drug reaction; *SD* standard deviation

**Supplementary Table 2. Treatment taken after the onset of ADRs of safety specifications (incidence ≥5%) and the outcomes**

| **Treatment taken after the onset of ADRs**  **(including an overlapping)** | **n** | **(%)** | **Number of events** | **Outcomes (number of events)** | | | | | |
| --- | --- | --- | --- | --- | --- | --- | --- | --- | --- |
|  |  |  |  | **Recovered** | **Recovering** | **Not recovered** | **Recovered**  **with sequelae** | **Death** | **Unknown** |
| Treatment for hepatic impairment | | | | | | | | | |
| Total | 16 | (100.00) | 17 | 11 | 6 | 0 | 0 | 0 | 0 |
| No treatment taken (observation only) | 3 | (18.75) | 3 | 2 | 1 | 0 | 0 | 0 | 0 |
| Dosage adjusted for dabrafenib | 5 | (31.25) | 5 | 5 | 0 | 0 | 0 | 0 | 0 |
| Dosage adjusted for trametinib | 4 | (25.00) | 4 | 4 | 0 | 0 | 0 | 0 | 0 |
| Interrupted dabrafenib | 0 | - | 0 | 0 | 0 | 0 | 0 | 0 | 0 |
| Interrupted trametinib | 0 | - | 0 | 0 | 0 | 0 | 0 | 0 | 0 |
| Interrupted dabrafenib and trametinib | 9 | (56.25) | 10 | 6 | 4 | 0 | 0 | 0 | 0 |
| Discontinuation of dabrafenib | 1 | (6.25) | 1 | 1 | 0 | 0 | 0 | 0 | 0 |
| Discontinuation of trametinib | 1 | (6.25) | 1 | 1 | 0 | 0 | 0 | 0 | 0 |
| Pharmacological treatment | 4 | (25.00) | 4 | 1 | 3 | 0 | 0 | 0 | 0 |
| Non-pharmacological treatment | 0 | - | 0 | 0 | 0 | 0 | 0 | 0 | 0 |
| Hospitalization or hospitalization prolonged | 2 | (12.50) | 2 | 2 | 0 | 0 | 0 | 0 | 0 |
| Treatment for pyrexia | | | | | | | | | |
| Total | 49 | (100.00) | 59 | 36 | 19 | 4 | 0 | 0 | 0 |
| No treatment taken (observation only) | 8 | (16.33) | 8 | 6 | 1 | 1 | 0 | 0 | 0 |
| Dosage adjusted dabrafenib | 5 | (10.20) | 5 | 3 | 2 | 0 | 0 | 0 | 0 |
| Dosage adjusted trametinib | 4 | (8.16) | 4 | 3 | 1 | 0 | 0 | 0 | 0 |
| Interrupted dabrafenib | 1 | (2.04) | 1 | 0 | 1 | 0 | 0 | 0 | 0 |
| Interrupted trametinib | 0 | - | 0 | 0 | 0 | 0 | 0 | 0 | 0 |
| Interrupted dabrafenib and trametinib | 14 | (28.57) | 16 | 13 | 3 | 0 | 0 | 0 | 0 |
| Discontinuation of dabrafenib | 3 | (6.12) | 3 | 2 | 1 | 0 | 0 | 0 | 0 |
| Discontinuation of trametinib | 4 | (8.16) | 4 | 3 | 1 | 0 | 0 | 0 | 0 |
| Pharmacological treatment | 36 | (73.47) | 42 | 22 | 17 | 3 | 0 | 0 | 0 |
| Non-pharmacological treatment | 1 | (2.04) | 1 | 1 | 0 | 0 | 0 | 0 | 0 |
| Hospitalization or hospitalization prolonged | 10 | (20.41) | 10 | 8 | 2 | 0 | 0 | 0 | 0 |

| Treatment for eye disorders | | | | | | | | | |
| --- | --- | --- | --- | --- | --- | --- | --- | --- | --- |
| Total | 6 | (100.00) | 8 | 1 | 5 | 1 | 0 | 0 | 1 |
| No treatment taken (observation only) | 0 | - | 0 | 0 | 0 | 0 | 0 | 0 | 0 |
| Dosage adjusted dabrafenib | 3 | (50.00) | 4 | 1 | 2 | 1 | 0 | 0 | 0 |
| Dosage adjusted trametinib | 1 | (16.67) | 2 | 0 | 1 | 1 | 0 | 0 | 0 |
| Interrupted dabrafenib | 0 | - | 0 | 0 | 0 | 0 | 0 | 0 | 0 |
| Interrupted trametinib | 0 | - | 0 | 0 | 0 | 0 | 0 | 0 | 0 |
| Interrupted dabrafenib and trametinib | 2 | (33.33) | 2 | 0 | 2 | 0 | 0 | 0 | 0 |
| Discontinuation of dabrafenib | 3 | (50.00) | 3 | 0 | 2 | 0 | 0 | 0 | 1 |
| Discontinuation of trametinib | 3 | (50.00) | 3 | 0 | 2 | 0 | 0 | 0 | 1 |
| Pharmacological treatment | 1 | (16.67) | 2 | 0 | 2 | 0 | 0 | 0 | 0 |
| Non-pharmacological treatment | 0 | - | 0 | 0 | 0 | 0 | 0 | 0 | 0 |
| Hospitalization or hospitalization prolonged | 1 | (16.67) | 1 | 0 | 1 | 0 | 0 | 0 | 0 |

*ADR* adverse drug reaction
